# Resveratrol Enhances Cytotoxic Effects of Cisplatin by Inducing Cell Cycle Arrest and Apoptosis in Ovarian Adenocarcinoma SKOV-3 Cells through Activating the p38 MAPK and Suppressing AKT

**DOI:** 10.3390/ph16050755

**Published:** 2023-05-17

**Authors:** Phateep Hankittichai, Phatarawat Thaklaewphan, Nitwara Wikan, Jirapak Ruttanapattanakul, Saranyapin Potikanond, Duncan R. Smith, Wutigri Nimlamool

**Affiliations:** 1 Department of Pharmacology, Faculty of Medicine, Chiang Mai University, Chiang Mai 50200, Thailand; phateep.han@cmu.ac.th (P.H.); phatarawat.th@gmail.com (P.T.); nitwara.wik@cmu.ac.th (N.W.); jirapak.ken@gmail.com (J.R.); saranyapin.p@cmu.ac.th (S.P.); 2 Research Center of Pharmaceutical Nanotechnology, Faculty of Pharmacy, Chiang Mai University, Chiang Mai 50200, Thailand; 3 Institute of Molecular Biosciences, Mahidol University, Salaya, Nakhon Pathom 73170, Thailand

**Keywords:** resveratrol, ovarian clear cancer, ovarian adenocarcinoma, SKOV-3 cells, p38 MAPK activation, PI3K/AKT

## Abstract

In the current study, we identified a mechanism of resveratrol (RES) underlying its anti-cancer properties against human ovarian adenocarcinoma SKOV-3 cells. We investigated its anti-proliferative and apoptosis-inducing effects in combination with cisplatin, using cell viability assay, flow cytometry, immunofluorescence study and Western blot analysis. We discovered that RES suppressed cancer cell proliferation and stimulated apoptosis, especially when combined with cisplatin. This compound also inhibited SKOV-3 cell survival, which may partly be due to its potential to inhibit protein kinase B (AKT) phosphorylation and induce the S-phase cell cycle arrest. RES in combination with cisplatin strongly induced cancer cell apoptosis through activating the caspase-dependent cascade, which was associated with its ability to stimulate nuclear phosphorylation of p38 mitogen-activated protein kinase (MAPK), well recognized to be involved in transducing environmental stress signals. RES-induced p38 phosphorylation was very specific, and the activation status of extracellular signal-regulated kinase 1/2 (ERK1/2) and c-Jun N-terminal kinase (JNK) was not mainly affected. Taken together, our study provides accumulated evidence that RES represses proliferation and promotes apoptosis in SKOV-3 ovarian cancer cells through activating the p38 MAPK pathway. It is interesting that this active compound may be used as an effective agent to sensitize ovarian cancer to apoptosis induced by standard chemotherapies.

## 1. Introduction

Ovarian cancer is a well-known lethal gynecological cancer that impacts females worldwide [1]. Most cases of ovarian cancer are typically due to epithelial cells and are divided into five subtypes: high-grade serous, low-grade serous, mucinous, endometrioid, and clear cell carcinoma [2]. Among ovarian epithelial cells, high-grade serous carcinoma is the most common subtype in ovarian cancer patients [3,4]. Between 2007 and 2013, the 5-year cause-specific survival of patients diagnosed with stage III and stage IV serous carcinomas was 42% and 26%, respectively [5]. Although most patients respond well to conventional chemotherapies, some gradually acquire chemoresistance during the remedy and eventually experience relapse or recurrence [6,7,8]. SKOV-3, a high-grade serous ovarian cancer cell line, has been reported to contain many factors that contribute to cancer survival and chemoresistance, including the absence of the p53 protein [9,10,11], mutation of adenine/thymine (AT)-rich interactive domain-containing protein 1A (ARID1A) [12], mutation of phosphatidylinositol-4,5-bisphosphate 3-kinase catalytic subunit alpha (PIK3CA) genes [13], and overexpression of histone deacetylase isoform [14]. Mitogen-activated protein kinase (MAPK) pathways are well-known kinase modules that indeed link with cancer and play a crucial role in transducing the signal from the environment to the cell for regulating the fundamental cell process, including proliferation, differentiation, migration, and apoptosis [15]. MAPKs comprise six subgroups: extracellular signal-regulated kinase (ERK)1/2, ERK3/4, ERK5, ERK7/8, Jun N-terminal kinase (JNK)1/2/3, and p38. p38 is one of the stress-activated MAPK, which mainly responds to inflammation, DNA damage, and apoptosis [16]. In cancers, its effect depends on the cellular context; p38 can promote tumorigenesis and cell migration in breast cancer [17,18]. However, abundant evidence confirms its anti-proliferative and pro-apoptotic effects, and a decrease in phosphorylated p38 is associated with chemoresistance in various cancers [19,20,21]. Hence, the discovery of new approaches that can enhance chemotherapy’s effect on ovarian cancer homeostasis is interesting since it may give better clinical outcomes, lower the side effects of chemotherapy, and increase the quality of life during treatment.

Resveratrol (trans-3′,4,5′-trihydroxystilbene, RES) is a natural polyphenolic stilbene primarily found in red wine, grapes, and berries [22]. It possesses diverse pharmacological activities, including antimicrobial, antioxidant, neuroprotective, anti-aging, anti-inflammatory, cardioprotective, blood-sugar-lowering, estrogenic/antiestrogenic, and anti-cancer activities [23,24,25,26,27,28]. Particularly, considering its anti-cancer effect, RES has been reported to induce programmed cell death in various types of human cancer cells including ovarian cancer cells [29]. Several previous studies have revealed that RES activates ERKs, JNKs, and p38 kinases along with inducing p53 phosphorylation that triggers cell apoptosis in mouse JB6 epidermal cell lines [30]. In cisplatin-resistant human oral squamous cell carcinoma (CAR) cell lines, non-toxic doses of RES reduced matrix metalloproteinase (MMP) 2 and 9 activities, suggesting that RES suppresses the metastatic property of cancer and keeps cancer at its origin site [31]. Moreover, RES inhibited AKT/mTOR phosphorylation while activating p38-MAPK to induce autophagy in non-small-cell lung cancer cell lines (A549 and H1299) [32]. To improve the p38 MAPK targeted therapy, we focused on this intriguing natural compound since it represents outstanding potency to stimulate p38 activation and induce cell apoptosis. Therefore, the current study elucidated the functional effects of RES in combination with cisplatin on inducing apoptotic cell death as well as explored their regulatory effects on p38-MAPK, PI3K/AKT, and other important signaling pathways in human ovarian cancer cells, SKOV-3. We believe that RES is an excellent candidate to be developed as a promising agent targeting p38 MAPK and AKT for ovarian cancer therapy.

## 2. Results

### 2.1. RES in Combination with Cisplatin Effectively Suppresses SKOV-3 Ovarian Cancer Cell Growth and Viability

**Figure 1:**
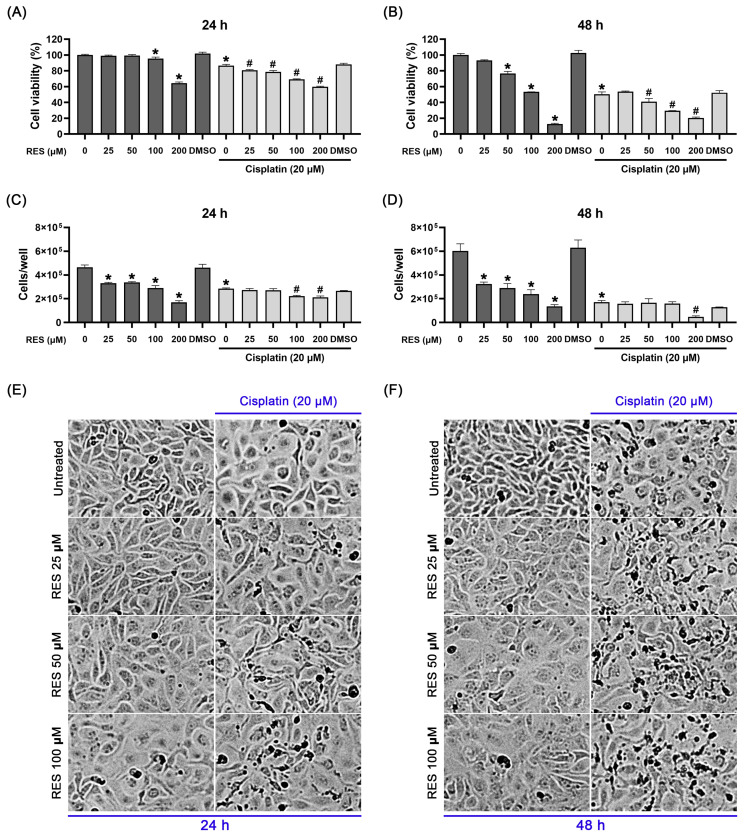
Effects of resveratrol (RES) on cell viability and proliferation of SKOV-3 ovarian cancer cells. Percent viability of cell treated with RES (25, 50, 100 and 200 µM) alone or in combination with cisplatin (20 µM) for 24 h (**A**) and 48 h (**B**). The number of cells quantified after treatment with RES (25, 50 and 200 µM) alone or in combination with cisplatin (20 µM) for 24 h (**C**) and 48 h (**D**). Cell morphological changes of SKOV-3 cells taken using a bright-field microscope at 24 h (**E**) and 48 h (**F**). Values represent mean ± SD of three independent experiments. * *p* < 0.05 vs. the untreated cells. # *p* < 0.05 vs. the cisplatin-treated cells.

To determine the effects of RES on the growth of ovarian cancer cells, SKOV-3 cells were initially treated with different concentrations of RES in complete media for 24 and 48 h prior to the MTT assay. In Figure 1A, we observed that the incubation of SKOV-3 cells with RES alone for 24 h caused a reduction trend in cell viability, with the effects of RES at 50 and 100 µM being significantly different. Continuing RES treatment for 48 h resulted in a concentration-dependent decrease in cell viability, and the half maximal inhibitory concentration (IC_50_) was determined to be 126.72 µM. To further address whether RES can enhance the effect of cisplatin, we thus performed viability and counting assays in cells co-treated with cisplatin and RES. We first tested the cytotoxicity of cisplatin at various concentrations (0-80 µM) on SKOV-3 cells. We found that cisplatin induced morphological changes and reduced SKOV-3 cell viability in a dose-dependent manner, and its IC50 was approximately 20 µM (Figure S1, Supplementary Materials). Based on this information and previous studies reporting that cisplatin at 20 µM efficiently induces DNA damage and apoptotic death in SKOV-3 cell lines [33,34,35], we thus utilized cisplatin at 20 µM in our study. We demonstrated that cisplatin alone could significantly decrease the cell viability to about 90% and 50% after 24 h and 48 h, respectively (Figure 1A,B). Additionally, after 24 h of co-treatment with 20 µM cisplatin in combination with RES (25, 50, 100 and 200 µM), cell viability significantly reduced, with the maximal inhibition (about 40%) being seen in cells treated with cisplatin plus 200 µM of RES (Figure 1A). As shown in Table S1 (Supplementary Materials), the synergy quotient (SQ) calculation of cell viability at 24 h indicated that a synergistic effect was found when 20 µM cisplatin was applied to the treatment of RES at 25, 50 and 100 μM where the SQ values were 1.34, 1.51 and 1.71, respectively. These results reveal the synergistic effects of combination treatments of cisplatin with RES at this range of concentrations. However, the SQ value of cells treated with cisplatin and RES at 200 μM was 0.82, indicating no synergism between the two agents. After 48 h, cisplatin treatment in combination with RES at 50, 100 and 200 µM significantly reduced cell viability to around 40, 30 and 20%, respectively, compared to that of cisplatin treatment alone (50%) (Figure 1B). These results suggest that RES has inhibitory effects on the cancer cell proliferation. Although RES–cisplatin co-treatment significantly enhanced the inhibition of cell viability, we observed that the SQ values of the co-treatment with cisplatin and RES at 25, 50, 100 and 200 µM were 0.82, 0.81, 0.73 and 0.58, respectively, indicating no synergism between the two agents at this time point.

To support the hypothesis that RES may have inhibitory effects on cancer cell proliferation, we conducted a direct counting experiment to assess the changes in cell density at 24 and 48 h post-treatment. Results indicated that treatment with RES alone at 25, 50 and 100 µM for 24 and 48 h could clearly lower the number of SKOV-3 cells over time, and the inhibitory effects were observed to increase along with the increased concentrations (Figure 1C,D). Moreover, the reduction in the cell number was enhanced with the presence of cisplatin. Based on these findings, we hypothesized that RES may promote SKOV-3 ovarian cell death, especially when it is combined with cisplatin. Thus, we performed an experiment to explore the effect of RES (with and without cisplatin) on the morphology of SKOV-3 cells after 24 and 48 h of treatment. As shown in Figure 1E, the untreated cells showed normal characteristics of adhesive epithelia, and treatment with RES alone at all three concentrations (25, 50 and 100 µM) for 24 h seemed to slightly increase the size of SKOV-3 cells. In contrast, the morphology of cisplatin-treated SKOV-3 cells was clearly altered. Specifically, their size was relatively larger, causing a lower degree of cell density. RES at 25 µM caused no significant alteration in the cellular appearance of cisplatin-treated cells. Interestingly, RES at 50 µM caused some population of cisplatin-treated cells to detach from the surface of the culture dish, and 100 µM of RES resulted in a drastic increase in this cell population, which exhibited blebbing appearances and an altered pattern of light reflection, suggesting that the cancer cells are undergoing cellular stress and apoptosis. Furthermore, the changes in cell morphology after treatment with RES or cisplatin–RES were observed to be clearly enhanced after 48 h of treatment whereas the untreated cells exhibited an increase in cell density, suggesting an increase in cell proliferation (Figure 1F).

### 2.2. RES Amplifies SKOV-3 Cell Death through Enhancing Caspase-Dependent Apoptosis

**Figure 2:**
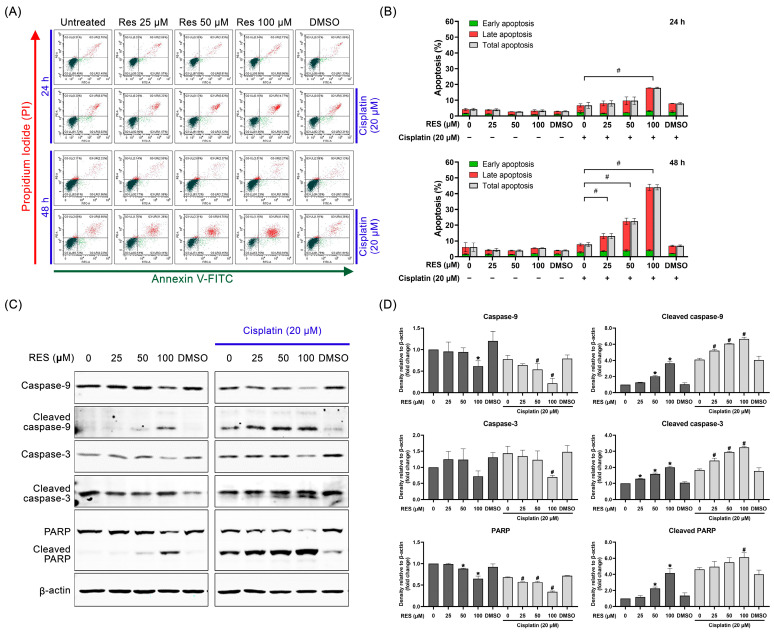
RES enhances cisplatin-induced SKOV-3 apoptosis. SKOV-3 cells were treated with RES (25, 50 and 100 μM) alone or in combination with cisplatin (20 μM) for 24 h or 48 h. (**A**) Annexin V/PI double staining and flow cytometry were performed for apoptosis analysis. (**B**) The right graphs show the quantification (%) of total, early and late apoptosis according to treatment at each time point; values represent mean ± SD of three independent experiments. # *p* < 0.05 vs. cisplatin-treated cells. (**C**) Western blot analysis, detecting caspase-9, caspase-3, PARP, and their cleaved forms, was performed as described in the ‘Materials and Methods’; β-actin was used to normalize protein expression. (**D**) Protein quantification via densitometry; values represent mean ± SD of three independent experiments. * *p* < 0.05 vs. untreated cells; # *p* < 0.05 vs. cisplatin-treated cells.

We further investigated whether RES enhances the effects of cisplatin on inducing cell apoptosis. To achieve this hypothesis, annexin V/PI was used to stain the apoptotic cells that were determined using flow cytometry. Results demonstrated that cisplatin–RES co-treatment created an increased trend of apoptotic cell death after 24 h, with cisplatin plus RES at 100 µM being significantly different (Figure 2A,B). As anticipated, after 48 h of co-treatment, a significant increase in cell apoptosis was observed in a dose-dependent manner (Figure 2A,B). In particular, cisplatin treatment in combination with RES at 25, 50 and 100 µM increased apoptotic cell death to approximately 10, 20 and 40%, respectively (Figure 2B). As shown in Table S2, the SQ calculation of cell apoptosis at 24 h indicated that a synergistic effect was found when cells were exposed to cisplatin plus RES at 50 and 100 μM. Furthermore, when cells were exposed to the co-treatment for 48 h, we observed the synergistic effects of combination treatments at all concentrations of RES, with 25 μM generating the minimal values of the synergy quotient. Consistently, results from Western blot analysis revealed that cisplatin–RES co-treatment for 48 h significantly enhanced the cleavage of caspases-9 and caspase-3. Likewise, poly (ADP-ribose) polymerase (PARP) protein, a major downstream substrate of executive caspase enzymes was progressively activated in response to cisplatin-RES co-treatment (Figure 2C). Densitometric analysis demonstrated that the total form of caspase 9, caspase 3, and PARP was gradually reduced in correlation with a significant increase in their cleaved forms in a dose-dependent manner when the cells were treated with cisplatin at 20 µM in combination with increasing concentrations of RES (Figure 2D).

### 2.3. RES Arrests SKOV-3 Cell Cycle in the S-Phase

**Figure 3:**
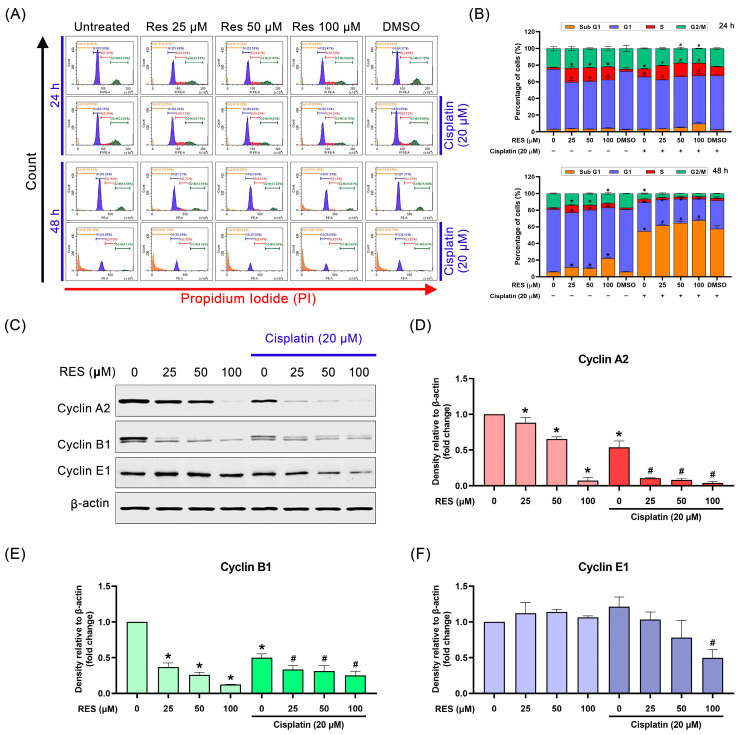
Effects of RES on cell cycle distribution of SKOV-3 cells. SKOV-3 cells were treated with RES (25, 50, and 100 µM) alone or in combination with cisplatin (20 µM) for 24 h and 48 h. (**A**) Cell cycle analysis was performed via flow cytometry as described in the Section 4. (**B**) The right graph shows the percent of the cell population at each stage of the cell cycle; values represent mean ± SD of three independent experiments. * *p* < 0.05 vs. untreated cells; # *p* < 0.05 vs. cisplatin-treated cells. (**C**) Western blot analysis of cyclin A2, cyclin B1, and cyclin E1 expression was performed as described in the ‘Materials and Methods’; β-actin was used to normalize protein expression. (**D**) The quantification of cyclin A2 protein expression assayed via densitometry. (**E**) The quantification of cyclin B1 protein expression assayed via densitometry. (**F**) The quantification of cyclin E1 protein expression assayed via densitometry. Values represent mean ± SD of three independent experiments. * *p* < 0.05 vs. untreated cells; # *p* < 0.05 vs. cisplatin-treated cells.

We conducted cell cycle assay via flow cytometry to determine the inhibitory activities of RES on the cancer cell proliferation (with and without co-treatment with cisplatin). We discovered that RES treatment (at 25, 50 and 100 µM) for 24 h significantly decreased the percentage of cells in the G1-phase (from approximately 70% to approximately 50%) and significantly elevated the percentage of cells in the S-phase (from approximately 2% to approximately 14%), but the treatment did not affect cell distribution in the Sub G1 or the G2-M phases (Figure 3A,B 24 h). Cisplatin treatment (alone) for 24 h also reduced the G1-phase population (from approximately 70% to approximately 60%) and increased the S-phase population (from approximately 2% to approximately 9%) without affecting the cell distribution in the Sub G1 or the G2-M phases (Figure 3A,B 24 h). Cisplatin–RES co-treatment for 24 h created similar patterns of cell cycle distribution to those of RES-treated cells, but it exhibited a rising trend in the Sub G1 population when the concentration of RES was increased (Figure 3A,B 24 h). Cell cycle analysis after 48 h of treatment demonstrated that there was no change in the cell cycle distribution in the untreated cells (compared to the untreated cells at 24 h), but RES-treated cells showed a significant decrease in the G1 population and a significant increase in the Sub G1 population (Figure 3A,B 48 h). Cisplatin treatment for 48 h drastically increased the Sub G1 population, and RES significantly enhanced the generation of this Sub G1 population in a concentration-dependent manner (Figure 3A,B 48 h). Based on these observations, we designed the experiment to monitor the effect of RES and cisplatin–RES on the molecular regulators of cell cycle progress. Since the activation of specific cyclin-dependent kinases is required for progression through the cell cycle, we then performed Western blot analysis to confirm that RES can genuinely arrest the cancer cell at the S-phase of the cell cycle. As shown in Figure 3C, RES treatment caused significant reduction in the level of cyclin A2 and cyclin B1 expression. In particular, RES at 25, 50 and 100 µM decreased expression of cyclin A2 by 11.62%, 34.59% and 92.73%, respectively, and cyclin B1 by 63.15%, 73.90% and 87.53%, respectively compared to the untreated cells (Figure 3D,E). Moreover, when the treatment was conducted with the presence of cisplatin, significantly enhanced reduction in cyclin A2 and B1 level was observed in a dose-dependent manner (Figure 3D,E). Although RES or cisplatin alone did not generate a significant change in cyclin E1 expression, we observed a decreasing trend in protein expression when cisplatin-treated cells were incubated with RES at increasing concentrations, especially at 100 µM (Figure 3A,F).

### 2.4. RES Specifically Induces p38 MAPK Activation and Suppresses AKT Activation

**Figure 4:**
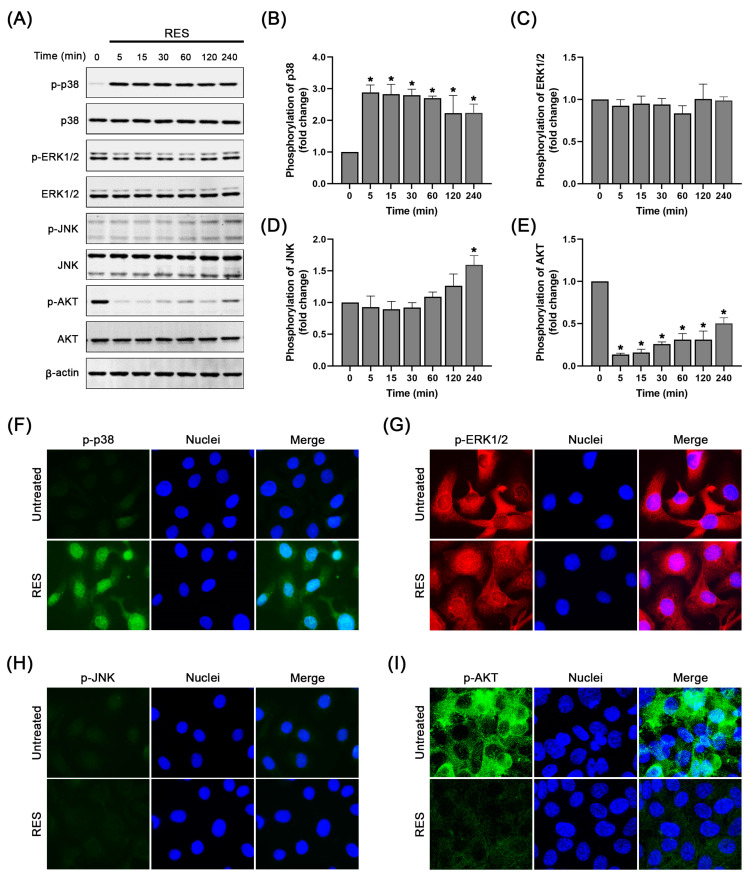
Effects of RES on regulating the MAPKs and PI3K/AKT pathways in SKOV-3 cells. (**A**) Western blot analysis of p-p38, total p38, p-ERK1/2, total ERK1/2, p-JNK, total JNK, p-AKT and total AKT in cells treated with 25 µM of RES at various time points (0–240 min). β-actin was shown as a loading control. Quantification of p-p38 (**B**), p-ERK1/2 (**C**), p-JNK (**D**) and p-AKT (**E**) upon treatment with 25 µM of RES at various time points (0–240 min). Values represent mean ± SD of three independent experiments. * *p* < 0.05 vs. RES-treated cells at 0 min. Immunofluorescence study of intracellular p-p38 (green) (**F**), p-ERK1/2 (red) (**G**), p-JNK (green) (**H**) and p-AKT (green) (**I**) in RES (25 µM)-treated cells. Nuclei were counterstained with DAPI (blue) (100× magnification).

Since various signaling molecules, such as MAPKs and PI3K/AKT pathways, have positive influences on cell growth and survival, we therefore examined a possible underlying mechanism of RES responsible for inhibiting the growth and survival of SKOV-3 ovarian cancer cells. The results from cell death and cell cycle assays, showing that the lowest concentration of RES (25 µM) could sufficiently induce cell cycle arrest and caspase activation (especially in combination with cisplatin), suggest that the compound has a potent ability to regulate the specific signal transduction pathways. Based on this information and the principal of rational drug approach that normally prefers selecting the lowest effective dose that still holds the potential for enhancing therapeutic outcomes, we thus firstly evaluated the regulatory effect of RES at 25 µM on different signal transduction pathways. Data from Western blot analysis showed that RES robustly induced p38 phosphorylation, which was about three-fold, at all-time points over 240 min, compared to that of the untreated cells. However, the phosphorylation of ERK1/2 was not induced (Figure 4A,C). The phosphorylation of stress-activated protein kinase (SAPK)/JNK, which is a kinase responsive to stress stimuli, was slightly increased (around 0.5 fold) at the 4 h time point (Figure 4A,D). Interestingly, the high basal level of AKT auto-phosphorylation in SKOV-3 cells was strongly suppressed by RES exposure over the course of the incubation time without affecting the expression level of AKT kinase (Figure 4A,E).

**Figure 5:**
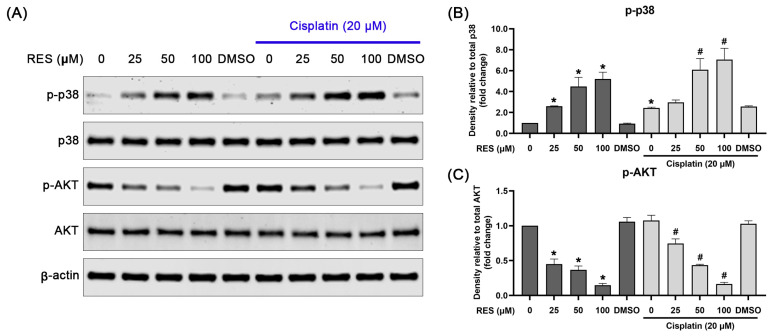
Dose-dependent effects of RES in combination with cisplatin on the phosphorylation of p38-MAPK and AKT in SKOV-3 cells. (**A**) Western blot detecting phosphorylation of p38 (p-p38), total p38 expression, phosphorylation of AKT (p-AKT) and total AKT expression after RES treatment (alone or in combination with cisplatin). Densitometric analysis of p-p38 (**B**) and p-AKT (**C**) (relative to their total kinase expression). Values represent mean ± SD of three independent experiments. * *p* < 0.05 vs. untreated cells. # *p* < 0.05 vs. RES with cisplatin-treated cells.

To ensure that RES has the specific effects on modulating p-38 MAPK phosphorylation, immunofluorescence analysis was further performed to visualize the localization pattern and strength of the activation signal in the cells. Consistent with the findings from Western blot analysis, treating SKOV-3 cells with RES dramatically stimulated p38 phosphorylation in the cell nuclei (Figure 4F). Nevertheless, RES treated-SKOV-3 cells exhibited no substantial change in the activation status of both ERK1/2 an JNK kinases (Figure 4G,H). In contrast, RES dramatically abolished the basal level of AKT phosphorylation (Figure 4I). These results suggest that RES mainly induces the activation status of p38 and suppresses AKT activation. Hence, we further evaluated the regulatory effects of RES on p38 and AKT at increasing concentrations, with and without the presence of cisplatin. As shown in Figure 5A,B, phosphorylation of p38 was significantly increased to around 3, 4 and 5 fold in cells treated with RES at 25, 50 and 100 µM, respectively. In contrast, the treatment caused a dose-dependent reduction in AKT phosphorylation. Treatment with cisplatin at 20 µM alone slightly induced p38 phosphorylation to about 2 fold, whereas treatment in combination with RES at 50 and 100 µM exhibited significant increases in p-p38 to approximately 6 and 7 fold, respectively. Interestingly, cisplatin treatment alone did not significantly suppress the basal level of p-AKT, and co-treatment of RES with cisplatin did not enhance the inhibitory effect of RES on p-AKT.

## 3. Discussion

Although there have been attempts to develop advanced treatment options for ovarian cancer, this type of cancer still has high incidence and mortality rates [36]. This may be due to the delayed diagnosis since ovarian cancer causes no specific symptoms in its early stages. Once it is diagnosed, ovarian cancer would have metastasized throughout the abdominal cavity, which may be one of the primary reasons why it is difficult to treat and has high mortality rates [37]. Similar to many other gynecologic cancers such as cervical cancer, screening and early detection diagnosis are effective means of ovarian cancer control. However, these aspects may not be practical for low-and middle-income countries where most people do not have access to the screening program. Standard treatments for ovarian cancer including surgical resection and platinum-based chemotherapy have been applied for patients, and these treatments can improve the prognosis of patients to a certain extent [38]. Nevertheless, it has been estimated that after surgery, the rate of ovarian cancer recurrence can be as high as 85%, with the 5-year survival rate being less than 30% [39,40]. Moreover, resistance to cisplatin is often observed in many patients, resulting in treatment failure. Specifically, the rapid rate of DNA repair and complete inhibition of apoptotic cell death are major possible mechanisms underlying cisplatin resistance [41]. Importantly, many patients experience serious adverse effects of cisplatin, which restrict its therapeutic uses [42]. Therefore, developing novel strategies to enhance the efficacy of cisplatin, apply cisplatin at a lower dose to avoid side effects, or reverse cisplatin resistance may help improve the therapeutic outcomes as well as the long-term survival rates of patients with ovarian cancer.

In the recent past, there have been extensive studies of natural compounds for their potential use for the targeting and chemoprevention of ovarian cancer [43]. One of the major compounds is RES, which is common in various species of plants [44,45]. The modulatory effects on inflammatory signaling pathways of RES in association with ovarian cancer have been shown [46]. Another study strongly suggested that RES induces ovarian cancer cell death through stimulating autophagy and apoptosis [47]. In OVCAR-3 ovarian cancer cells, it was demonstrated that RES blocked protein kinase B/glycogen synthase kinase (AKT/GSK) and ERK activation, resulting in suppression of cell proliferation [48]. In the epithelial ovarian cancer cell line A2780, RES was proved to prevent the development of cisplatin resistance [49]. Moreover, RES was shown to effectively block lysophosphatidic acid (LPA)-induced hypoxia-inducible factor 1-alpha (HIF-1α) and vascular endothelial growth factor (VEGF) expression in OVCAR-3 and CAOV-3 ovarian cancer cells [50]. Moreover, there has been an interesting study reporting that RES at a concentration non-toxic to normal human fibroblasts effectively killed A2780-derived ovarian cancer stem cells [51].

In our current study, we attempted to identify a novel mechanism of RES in SKOV-3 cells, an epithelial ovarian cancer cell line. Moreover, as a step towards the possible use of RES for enhancing the efficacy of chemotherapy for ovarian cancer, we applied the combination approach to determine the potential synergistic effects of RES and cisplatin. Data from a cell viability assay demonstrated that RES or cisplatin alone reduced the percent cell viability to a certain degree. As anticipated, cisplatin treatment in combination with increased concentrations of RES significantly decreased the percent SKOV-3 cell viability in a concentration-dependent manner. These data indicate that combination treatment of cisplatin–RES can effectively reduce the cell metabolic activity. In particular, we found a gradual synergistic effect in cells with RES–cisplatin co-treatment for 24 h, but this effect was not observed after 48 h of the co-treatment. This can be explained by the fact that treatment with RES or cisplatin alone for longer times was so strong that they caused a great reduction in the cell number (possibly due to a significant increase in the dead cell population). Hence, it is reasonable to conclude that the synergistic effect was no longer observed at this time point. Consistent with these results, we found that the number of RES-treated cells was also reduced in a concentration-dependent manner. The reduction in the cancer cell number was enhanced when cisplatin was present. Additionally, we observed that RES treatment with increasing concentrations decreased the cell density and induced cell morphological changes where cells exhibited an increase in their size over the course of 48 h. These data help explain the possibility that the reduction in cell viability observed in the MTT assay may be, at least in part, caused by the inhibitory effect of cisplatin–RES treatment on ovarian cancer cell proliferation. Moreover, these data strongly suggest the possible induction of cell cycle arrest since the co-treatment obviously induced cell morphological changes over the course of 48 h where the density of cells was reduced in contrast to their increased size.

These results support previous studies claiming that RES can enhance the sensitivity to cisplatin treatment. The reduction in percent cell viability examined using the MTT assay was verified, via flow cytometry (annexin V/PI staining), to be mainly caused by the induction of cancer cell death through the apoptotic pathway where RES clearly enhanced cisplatin-induced apoptotic cell death, since our analysis revealed significant synergism of cisplatin–RES treatment. Moreover, results from Western blot analysis confirmed that the treatment of SKOV-3 with RES combined with cisplatin caused ovarian cancer cell apoptosis via a caspase-dependent pathway observed by a decrease in the full-length caspase-9, caspase 3, and PARP and an increased in their cleaved forms, indicating the activation of caspase proteolytic activity. Similar apoptotic cell death patterns were reported in our previous study where SKOV-3 and TOV-21G ovarian cancer cells and cervical cancer cell lines were exposed with *Kaempferia parviflora* extract which contains methoxyflavones as its major active compounds [52,53,54,55]. A similar concept of combinatorial treatment has been reported to overcome ovarian cancer drug resistance. For instance, the combination of the diphtheria toxin and TNF-alpha exhibited synergistic cytotoxicity effects in sensitive and resistant human ovarian tumor cell lines including SKOV-3 cells [56]. More recently, the combination of curcumin and RES significantly sensitized the epithelial ovarian cancer A2780 (cisplatin-sensitive cell line) and A2780-cis (cisplatin-resistant cell line) to cisplatin, thereby inhibiting ovarian cancer cell chemoresistance via inhibiting the PI3K/AKT/mTOR pathway [57]. In line with the study of this group, we discovered that RES strongly inhibited the phosphorylation of AKT in SKOV-3 cells. This finding indicates that RES can negatively manipulate the crucial survival signaling in ovarian cancer cells.

In addition to the aspect of survival and apoptosis induction, we demonstrated that RES alone could disrupt the cell cycle control of ovarian SKOV-3 cancer cells by downregulating cyclin A2 expression, thereby inducing S-phase arrest. Induction of G2/M arrest represented by a reduction of cyclin B1 level in SKOV-3 cancer cells was subsequently seen to be an effect of RES treatment. The combination of cisplatin and RES created a significant decrease in cyclin A2 and B1 expressions while the level of cyclin E1 was slightly affected. These results explain that RES and cisplatin mainly attenuate the expression of crucial molecular components of the cell cycle and consequently induce cell cycle arrest at the S-phase. In addition, the co-treatment strongly induced a significant increase in the number of cells in the Sub G1 phases in a dose-dependent manner, indicating effective cell death induction.

In breast and lung cancer cells, RES induced cancer cell senescence via enhancing p38 MAPK [58]. We hypothesized that RES may exert similar signaling pathways to interfere with normal cell cycle control and survival of ovarian cancer cells. Data from Western blot analysis clearly demonstrated that RES rapidly induced p38 MAPK phosphorylation, and the activation of this kinase was relatively stable over the course of 240 min. The activation of p38 MAPK was detected to be localized in the nuclei of RES-treated SKOV-3 cells. This finding indicates that RES can quickly induce stress, which then relays the signal to activate p38 MAPK phosphorylation. We also observed that the phosphorylation level of p-p38 was strongly increased with increased concentration of RES whereas AKT phosphorylation was dramatically declined. When RES was combined with cisplatin, the activation of p-p38 was slightly increased, but no enhanced inhibition of AKT activation was observed. We also detected the activation status of the other two MAP kinase enzymes (ERK1/2 and JNK) upon RES exposure but observed no strong change in the phosphorylation status of these kinases. These data suggest that RES may distinctly modulate these signal transduction pathways to enhance the cytotoxicity of cisplatin. It has been shown that an increase in p38 activity under the influence of transforming growth factor 2 (TGF2) suppressed the metastasis of human head and neck squamous cell carcinoma (HNSCC) cells, while the inhibition of p38 MAPK activity resulted in tumor proliferation [59]. RES was reported to induce p38 MAPK, which resulted in suppressed metastatic behaviors of cisplatin-resistant human oral cancer cells [31]. Our findings are consistent with this report where enhanced cytotoxic effects of RES on ovarian cancer cell upon cisplatin treatment can be explained by its ability to maintain the robust activation of p38 MAPK (while suppressing AKT activation), making the cancer cells more susceptible to apoptotic death induction. In addition, our observation that RES disrupted the SKOV-3 ovarian cancer cell cycle is supported by the evidence that p38 MAPK regulates the proliferation at both the G1/S and G2/M phases of the cell cycle by activating checkpoint responses [60].

**Figure 6:**
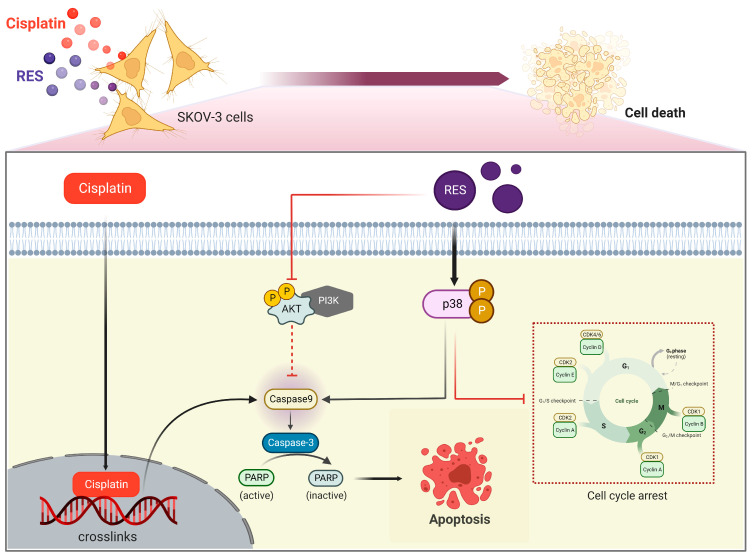
Schematic image proposing the mechanism that resveratrol (RES) triggers cell cycle arrest and apoptosis of human ovarian cancer cells, SKOV-3. The illustration was created with BioRender.com (accessed on 15 May 2023).

In conclusion, as depicted in Figure 6, our study provided more accumulated evidence that RES increases the sensitivity of ovarian cancer cells to cisplatin, making them more susceptible to cell cycle arrest and apoptotic cell death. The effect of RES was proved to originate from its capability to specifically activate p38 MAPK and inhibit AKT activation. Despite these promising in vitro results and data from previous reports, RES has not yet been approved as a potential therapeutic agent for the treatment of epithelial ovarian cancer, since many aspects related to the potency and mechanism of action, especially when RES is combined with standard chemotherapy drugs, remain to be elucidated. Indeed, studies about how RES simultaneously induces intracellular stress signaling and suppresses growth/survival signal transduction pathways in SKOV-3 ovarian cancer cell line have not been elucidated. Our study highlights the potential role of RES in combination with cisplatin against ovarian cancer. In particular, we reported more in-depth data that RES possesses distinct pro-apoptotic activity for enhancing the cytotoxic effect of cisplatin through activating the activity of p38 MAPK while suppressing PI3K/AKT activation. This is beneficial for accelerating its clinical application, especially in case where the effects of chemotherapeutic drugs rely on p38 MAPK activation and PI3K/AKT suppression.

## 4. Materials and Methods

### 4.1. Cells and Reagents

Human ovarian adenocarcinoma cells (SKOV-3) were purchased from American Type Culture Collection (Manassas, VA, USA). RES was purchased from MilliporeSigma (Burlington, MA, USA). MTT reagent (3-(4,5-dimethylthiazol-2-yl)-2,5-diphenyltetrazolium bromide) was purchased from MilliporeSigma. Guava^®^ Cell Cycle Reagent was purchased from Luminex Corporate (Austin, TX, USA). Annexin V was purchased from ImmunoTools (Friesoythe, Niedersachsen, Germany). Propidium iodide was purchased from MilliporeSigma. Rabbit anti-phospho p38 antibody, rabbit anti p38 antibody, rabbit anti-phospho ERK1/2 antibody, mouse anti-ERK1/2 antibody, rabbit anti-phospho JNK antibody, rabbit anti-JNK antibody, rabbit anti-phospho AKT (Ser473) antibody, mouse anti-AKT antibody, rabbit anti-cyclin A2 antibody, rabbit anti-cyclin B1 antibody, rabbit anti-cyclin E1 antibody, rabbit anti-caspase-9 antibody, rabbit anti-caspase-3 antibody, rabbit anti-PARP antibody, mouse anti-β-actin antibody, and DAPI (4, 6-diamidino-2-phenylindole, dihydrochloride) were purchased from Cell Signaling Technology (Danvers, MA, USA). Goat anti-mouse IgG-IRDye^®^800CW and goat anti-rabbit IgG-IRDye^®^680RT were purchased from Li-COR Biosciences (Lincoln, NE, USA). Goat anti-rabbit conjugated with Alexa488 and Goat anti-mouse conjugated with Alexa594 were purchased from Thermo Fisher Scientific (Waltham, MA, USA).

### 4.2. Cell Culture and RES Treatment

SKOV-3 cells were cultured in RPMI 1640 (Gibco, BRL, USA), containing 10% fetal bovine serum (Merck KGaA, Darmstadt, Germany), 100 U/mL penicillin, and 100 g/mL streptomycin (both antibiotics from Thermo Fisher Scientific). The cells were incubated in a humid environment with a 37 °C temperature and 5% CO_2_ concentration. Every three days, the medium was replenished or changed. When cells were 90% confluent, they were subcultured. Lower doses of RES (6.25, 12.5 and 25 µM) were added to SKOV-3 cells for the appropriate amount of time in serum-free RPMI 1640 to examine the effect of RES on early cell signaling. In other experiments related to cell cycle arrest and cell death, RES at high doses (25, 50 and 100 µM) was used. For experiments that involved cisplatin treatment, cells were treated with cisplatin alone (20 µM) or the mixture of 20 µM cisplatin and RES at various concentrations (25, 59 and 100 µM) for 24 and 48 h.

### 4.3. Cell Viability Assay

In 96-well plates, SKOV-3 cells were seeded at 1.0 × 10^4^ cells/well in complete RPMI 1640 and incubated for 24 h. Then, cells were exposed to various doses of RES for 48 h. After that, cells were cultured for an additional hour with 200 µL of media containing MTT reagent at a final concentration of 0.4 mg/mL. The supernatant was then removed, and 100 µL of 100% DMSO was added to dissolve the MTT crystal. Colorimetric measurement at 570 nm was performed using a microplate reader (BioTek Instruments, Winooski, VT, USA).

### 4.4. Cell Counting

SKOV-3 cells at a density of 1.0 × 10^6^ cells/well in RPMI 1640 were seeded in 24-well plates and cultured for 24 h. The medium was replaced with a serum-free medium, and the cells were treated with RES at different concentrations (0, 5, 10 and 20 µM). The incubation time was designed to cover 2 days; cells were collected and counted at 0, 24 and 48 h. Cell collection was performed by washing adherent cells with 1× PBS and treating cells with 1X trypsin solution (100 µL) for 10 min before adding 400 µL of complete medium (RPMI 1640 with 10% FBS). The cell suspension in each well was counted three times by using the CellDrop™ Automated Cell Counter (DeNovix Inc., Wilmington, DE, USA). The experiment was repeated 3 times.

### 4.5. Cell Apoptosis Analysis by Annexin V/PI Staining

Cells were seeded in 24-well plates at a density of 0.2 × 10^6^ cells/well in RPMI 1640 for 24 h. The medium was replaced with a serum-free medium, and cells were cultured for 24 h. Next, cells were treated with RES at different concentrations (0, 25, 50 and 100 µM) with or without the presence of cisplatin (20 µM) for 48 h. After removing the medium, cells were trypsinized, centrifuged, and then washed once in PBS. Then, cell pellets were reconstituted in 1X annexin-binding buffer. Following the addition of annexin V and propidium iodide (PI), the cells were incubated at RT for 15 min. Using a Beckman Coulter DxFLEX flow cytometer (Brea, CA, USA), labeled cells were immediately examined via flow cytometry to measure the percentage of apoptotic cell death. Utilizing the CytExpert for the DxFLEX program, data analysis was carried out.

### 4.6. Cell Cycle Analysis via Flow Cytometry

SKOV-3 cells (0.2 × 10^6^ cells) were seeded in 24-well plates in RPMI 1640 for 24 h. The medium was altered to a serum-free medium, and cells were further cultured for 24 h. After that, various concentrations of RES (0, 25, 50 and 100 µM) were used to treat the cells with or without the presence of cisplatin (20 µM) for 24 h. The culture medium was removed, and cells were washed thrice with 1X PBS. Cells were collected via trypsinization for 10 min and washed once with 1X PBS using centrifugation. Cells were then simultaneously fixed and permeabilized using 70% cold ethanol in a dropwise manner on a proper vortex to prevent cell aggregation. Cells were fixed in ethanol for 24 h at −20 °C. Cells were washed once with 1X PBS via centrifugation, and incubated with 150 µL of Guava^®^ Cell Cycle Reagent in the dark for 15 min at RT. Stained cells were analyzed immediately via flow cytometry using a flow cytometer DxFLEX from Beckman Coulter. Data analysis was performed using the CytExpert for DxFLEX software (version 2.0.0.283), and results were presented as the ratio of cell population in the G1, S and G2/M phases.

### 4.7. Western Blot Analysis

SKOV-3 cells in RMPI 1640 were seeded in 24-well plates at a density of 0.2 × 10^6^ cells/well and cultured for 24 h. For early cell signaling detection, cells were treated with RES at 25 µM for different time points (0, 5, 15, 30, 60, 120 and 240 min). For studying the dose-response effect on signaling pathways in combination with cisplatin, cells were treated with RES alone at 25, 50 and 100 µM or RES at these concentrations with the presence of 20 µM cisplatin for 240 min. For cell-cycle check-point protein detection, cells were treated with RES at 25, 50 and 100 µM with or without the presence of cisplatin at 20 µM for 24 h. For pro-apoptotic protein detection, cells were treated with RES at 25, 50 and 100 µM with or without the presence of cisplatin at 20 µM for 48 h. Then, 150 µL of 1X reducing Laemmli buffer was added to harvest the cell lysates. After that, the cell lysates were heated at 95 °C for 5 min, separated via SDS-PAGE using 10% gels, and transferred onto polyvinylidene difluoride (PVDF) membranes (GE Healthcare, Chicago, IL, USA). After blocking with a blocking solution (5% bovine serum albumin (BSA) in TBST) for 1 h at room temperature (RT), membranes were incubated overnight with 1:1000 (diluted in blocking solution) of antibodies against phosphorylated p38 (Thr180/Tyr182), total p38, phosphorylated ERK1/2, total ERK1/2, phosphorylated JNK, total JNK, phosphorylated AKT (Ser473), total AKT, cyclin A2, cyclin B1, cyclin E1, caspase-9, caspase-3, PARP, and β-actin. Then, membranes were washed thrice with TBST (5 min each time) and immersed with appropriate secondary antibodies for 1 h. After washing the membranes thrice with TBST, immunoreactive bands were visualized and recorded with the Odyssey^®^ CLx Imaging System (LI−COR Biosciences). The band intensity was analyzed and quantified using the ImageJ software (version 1.51j8).

### 4.8. Immunofluorescence Study

A total of 0.5 × 10^6^ cells/well of SKOV-3 were seeded onto glass coverslips placed in 3 cm dishes and cultured for 24 h in complete RPMI 1640. The medium was then changed to serum-free medium, and cells were further cultured for 24 h. Then, cells were treated with RES at 25 µM for 15 min. Cells were washed once with 1X PBS and fixed with 4% paraformaldehyde for 15 min. Sample coverslips were washed 3 times with PBS (5 min each time) on an orbital shaker and then permeabilized with 0.3% TritonX-100 in PBS for 5 min. After washing thrice, sample coverslips were blocked with 1% BSA in PBS for 1 h at RT and incubated with primary antibodies in a moist chamber at 4 °C overnight. Sample coverslips were washed 3 times with 1X PBS and incubated with a secondary antibody conjugated with Alexa488 or Alexa594 for 2 h in a moist chamber at RT. Sample coverslips were washed 3 times with 1X PBS and 1 time with deionized water (5 min) before mounting with Fluoromount-G (SouthernBiotech, Birmingham, AL, USA). Signals of the target proteins were visualized at 100× magnification using a fluorescent microscope, Axio Vert.A1 (Carl Zeiss Suzhou Co., Ltd., Suzhou, China). The Zen version 2.6 (blue edition) software for the Zeiss Axiocam 506 color microscope camera was used to capture and analyze the images.

### 4.9. Statistical Analysis

Statistical analysis was carried out using The GraphPad Prism program version 9.0.0 (121) (GraphPad Software Inc., San Diego, CA, USA). Data are presented as the mean ± standard deviation (SD). Differences between groups were analyzed using one-way analysis of variance (ANOVA) with Tukey’s post hoc multiple comparisons on RAW data reads. Significant differences compared with appropriate controls are denoted with asterisks, * *p* (or ^#^ *p*) < 0.05. All experiments were repeated at least three times independently.

### 4.10. Synergy Quotient Calculation for Synergism

The synergism quotient (SQ) was determined by deducting the baseline values from all treatments and then dividing the net effect of the combination [A + B] by the sum of individual effects [A] + [B]. SQ greater than 1.0 reveals a synergistic effect.

## 5. Conclusions

The current study provides more accumulated evidence that RES stimulates p38-MAPK and suppresses AKT activation in ovarian cancer cells. RES slightly affects the activation status of other signaling molecules including ERK1/2 and JNK. These regulatory activities of RES enhance the effects of cisplatin on inducing cell cycle arrest via downregulating the expression of responsible cyclin proteins and inducing caspase activation, resulting in an increase in apoptotic cell death. Our discovery adds to the mechanism profile of RES, which may be beneficial for its potential uses in combination with standard platinum-based chemotherapies for ovarian cancer treatment.

## Data Availability

All data, tables and figures are original and are available in this article.

## Supplementary Materials

The following supporting information can be downloaded at: https://www.mdpi.com/article/10.3390/ph16050755/s1, Figure S1: Effect of cisplatin at varied concentrations (0-80 μM) for 48 h on SKOV-3 cell viability (determined by MTT assay), cell proliferation (assayed by direct cell counting), and cell morphological changes (examined by phase-contrast microscopy; Table S1: Synergism quotient of cisplatin on the RES-induced reduction in cell viability at 24 h and 48 h; Table S2: Synergism quotient of cisplatin on the RES-induced apoptosis at 24 h and 48 h.
