# Supplementary material for: Resveratrol Enhances Cytotoxic Effects of Cisplatin by Inducing Cell Cycle Arrest and Apoptosis in Ovarian Adenocarcinoma SKOV-3 Cells through Activating the p38 MAPK and Suppressing AKT
Source: Pharmaceuticals (Basel). 2023 May 17;16(5):755. doi: 10.3390/ph16050755 (PMC10220637; doi:10.3390/ph16050755)
Supplement: Supplementary file 1 [file pharmaceuticals-16-00755-s001.zip › pharmaceuticals-2310052-supplementary.pdf]

Supplementary Figure and Table

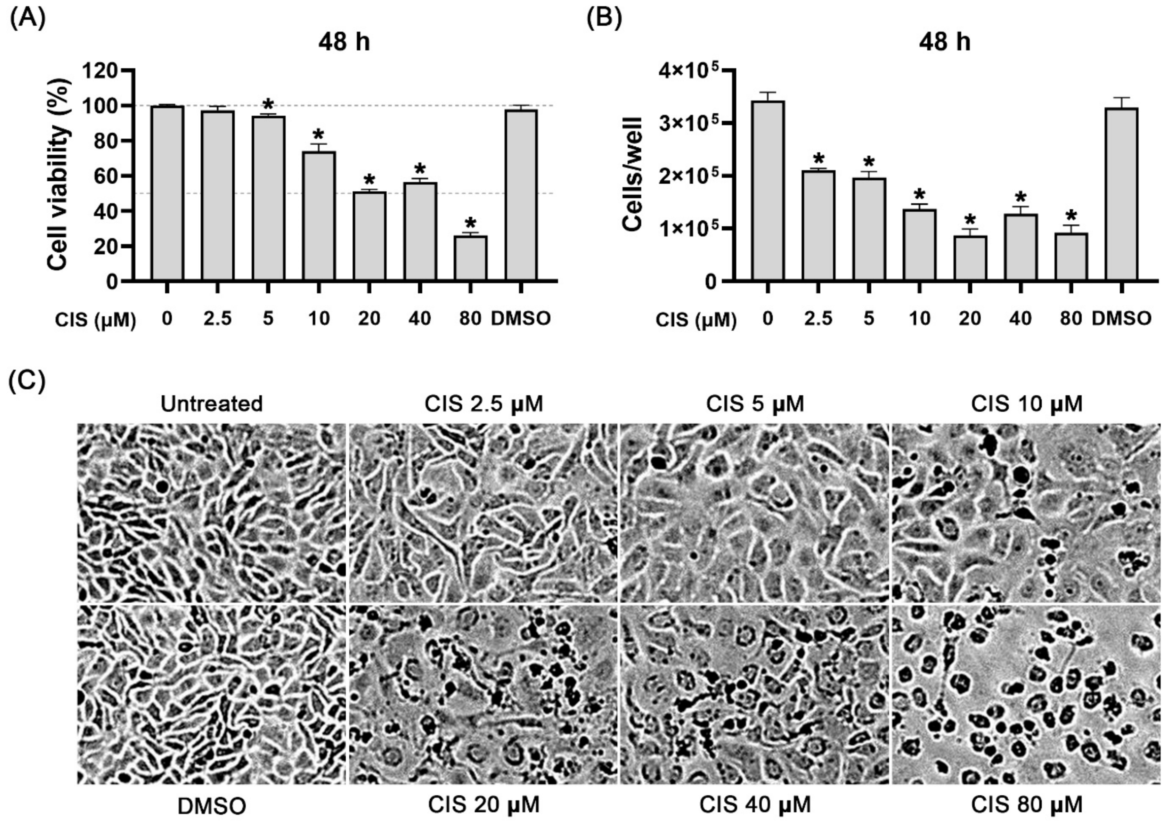

Figure S1. Effect of cisplatin at varied concentrations (0-80 μM) for 48 h on SKOV-3 cell viability (determined by MTT assay) (**A**), cell proliferation (assayed by direct cell counting) (**B**), and cell morphological changes (examined by phase-contrast microscopy (**C**). * $p < 0.05$ vs untreated cells

Table S1. Synergism quotient of cisplatin on the RES-induced reduction in cell viability at 24 h and 48 h

24 h	Cell viability (%)			Inhibition Rate	SQ Value
Cisplatin	86.55	±	1.57	13.45	
25 μM RES	99.11	±	0.72	0.89	
Cisplatin + 25 μM RES	80.83	±	0.72	19.17	1.34
50 μM RES	99.26	±	1.27	0.74	
Cisplatin + 50 μM RES	78.60	±	1.85	21.40	1.51
100 μM RES	95.39	±	1.95	4.61	
Cisplatin + 100 μM RES	69.17	±	0.65	30.83	1.71
200 μM RES	64.41	±	1.46	35.59	
Cisplatin + 200 μM RES	59.96	±	0.59	40.04	0.82
**48 h**	**Cell viability (%)**			**Inhibition Rate**	**SQ Value**
Cisplatin	50.28	±	3.03	49.72	
25 μM RES	93.14	±	0.80	6.86	
Cisplatin + 25 μM RES	53.78	±	0.64	46.22	0.82
50 μM RES	76.77	±	2.55	23.23	
Cisplatin + 50 μM RES	40.87	±	4.15	59.13	0.81
100 μM RES	53.40	±	0.10	46.60	
Cisplatin + 100 μM RES	29.56	±	0.10	70.44	0.73
200 μM RES	12.69	±	0.96	87.31	
Cisplatin + 200 μM RES	20.46	±	1.15	79.54	0.58

Table S2. Synergism quotient of cisplatin on the RES-induced apoptosis at 24 h and 48 h

**24 h**	**Apoptosis (%)**	**SQ Value**
Cisplatin	6.76 ± 1.89	
25 μM RES	3.94 ± 0.69	
Cisplatin + 25 μM RES	8.00 ± 1.70	0.75
50 μM RES	2.70 ± 0.08	
Cisplatin + 50 μM RES	9.67 ± 2.44	1.02
100 μM RES	3.26 ± 0.62	
Cisplatin + 100 μM RES	17.86 ± 0.47	1.78
**48 h**	**Apoptosis (%)**	**SQ Value**
Cisplatin	7.76 ± 1.31	
25 μM RES	4.27 ± 0.99	
Cisplatin + 25 μM RES	13.08 ± 1.61	1.09
50 μM RES	3.93 ± 0.40	
Cisplatin + 50 μM RES	22.52 ± 1.82	1.93
100 μM RES	5.47 ± 0.22	
Cisplatin + 100 μM RES	44.02 ± 1.60	3.33
